# Synaptic Vesicle Recycling Is Unaffected in the Ts65Dn Mouse Model of Down Syndrome

**DOI:** 10.1371/journal.pone.0147974

**Published:** 2016-01-25

**Authors:** Jamie R. K. Marland, Karen J. Smillie, Michael A. Cousin

**Affiliations:** Centre for Integrative Physiology, University of Edinburgh, Hugh Robson Building, 15 George Square, Edinburgh, EH8 9XD, United Kingdom; Flinders University, AUSTRALIA

## Abstract

Down syndrome (DS) is the most common genetic cause of intellectual disability, and arises from trisomy of human chromosome 21. Accumulating evidence from studies of both DS patient tissue and mouse models has suggested that synaptic dysfunction is a key factor in the disorder. The presence of several genes within the DS trisomy that are either directly or indirectly linked to synaptic vesicle (SV) endocytosis suggested that presynaptic dysfunction could underlie some of these synaptic defects. Therefore we determined whether SV recycling was altered in neurons from the Ts65Dn mouse, the best characterised model of DS to date. We found that SV exocytosis, the size of the SV recycling pool, clathrin-mediated endocytosis, activity-dependent bulk endocytosis and SV generation from bulk endosomes were all unaffected by the presence of the Ts65Dn trisomy. These results were obtained using battery of complementary assays employing genetically-encoded fluorescent reporters of SV cargo trafficking, and fluorescent and morphological assays of fluid-phase uptake in primary neuronal culture. The absence of presynaptic dysfunction in central nerve terminals of the Ts65Dn mouse suggests that future research should focus on the established alterations in excitatory / inhibitory balance as a potential route for future pharmacotherapy.

## Introduction

Down syndrome (DS) is the most common genetic cause of intellectual disability, and arises from the presence of an extra copy of human chromosome 21 (Hsa21) [1]. Patients with DS display reductions in synapse number [2], decreased dendrite arborisation [3,4] and an imbalance between excitatory and inhibitory input [5,6], suggesting synaptic dysfunction is a key factor in the disorder. Altered presynaptic function could underlie some of these perturbations, since several key endocytosis genes are present on Hsa21. Furthermore, enlarged early endosomes are observed in DS brain from before birth [7]. This may result from perturbed synaptic vesicle (SV) recycling, since evidence is accumulating that some SV endocytosis modes intersect with classical endosomal trafficking routes [8].

The activity-dependent fusion (exocytosis) and efficient retrieval (endocytosis) of SVs at the presynapse is essential for synaptic transmission, and disruption of these processes can result in a number of neurodevelopmental disorders [9]. Hsa21 carries genes for several proteins that are either proven (synaptojanin [10], intersectin [11,12], Dyrk1A [13,14]) or predicted (APP [15], RCAN1 [16]) to influence SV recycling in central nerve terminals. SVs are retrieved by three discrete endocytosis modes that are differentially recruited by neuronal activity. These are ultrafast endocytosis [17], clathrin-mediated endocytosis (CME, [18]) and activity-dependent bulk endocytosis (ADBE, [19]). CME forms single SVs directly from the plasma membrane and is the dominant SV endocytosis mode during mild activity [20]. During elevated neuronal activity ADBE is recruited to provide additional endocytic capacity. ADBE rapidly forms large bulk endosome structures directly from the plasma membrane, which generate SVs over time to replenish depleted pools [21]. The calcium-dependent protein phosphatase calcineurin (CaN) controls a number of events in the SV life cycle, including the number of available SVs [22], the speed of CME [23], triggering of ADBE [24,25] and SV generation from bulk endosomes [26]. In DS brains, increased expression of the endogenous CaN inhibitor RCAN1 [27,28] suggests these events could be specifically perturbed.

The Ts65Dn mouse model of DS [29] carries a segmental trisomy of mouse chromosome 16 (Mmu16), containing orthologues of approximately half the protein encoding genes on Hsa21 [30]. Comparative studies in trisomic neurons have highlighted increases in presynaptic and postsynaptic size [31], presence of enlarged endosomal structures [32], and alterations in neuronal intracellular trafficking [33]. The Ts65Dn mouse also shows disrupted synaptic plasticity, with decreased long-term potentiation (LTP) and increased long-term depression detectable in the hippocampus [34–37]. Importantly, behavioural assays show learning and memory are affected in these mice [38], strongly suggesting that the identified cellular defects are relevant to neurological problems in DS.

Considering the genetic and endosomal trafficking links to SV endocytosis, we investigated whether SV recycling was altered in the Ts65Dn mouse model. Using a combination of live imaging assays and ultrastructural analysis, we found that both CME and ADBE were unaffected in trisomic neurons. Thus, dysfunctional SV recycling is unlikely to precipitate the learning and memory defects in the Ts65Dn mouse, or by extension the neurological deficits that occur in DS.

## Methods

### Reagents & antibodies

All general reagents were from Sigma-Aldrich (Gillingham, UK), and all tissue culture reagents were from Life Technologies (Paisley, UK) unless otherwise stated. 6-Cyano-7-nitroquinoxaline-2,3-dione disodium salt (CNQX) and DL-2-amino-5-phosphonopentanoic acid sodium salt (AP5) were from Abcam (Cambridge, UK), and bafilomycin A1 was from Cayman Chemical (Ann Arbor MI, USA). Sheep anti-dynamin PSer774 and sheep anti-dynamin PSer778 were from AbD Serotec (Kidlington, UK), goat anti-dynamin I was from Santa Cruz (Dallas, USA), HRP-conjugated monoclonal mouse anti-β-actin (clone AC-15) and HRP-conjugated rabbit anti-sheep/goat were from Sigma-Aldrich. Donkey anti-goat IRDye 680RD and donkey anti-mouse IRDye 800CW were from LI-COR Biosciences (Cambridge, UK). The plasmid encoding synaptophysin-pHluorin (syp-pHluorin) was a gift from Prof. L. Lagnado (University of Sussex, UK).

### Mouse maintenance, breeding and genotyping

All founder mouse stocks were obtained from the Jackson Laboratory. C57BL/6JEiJ (Jax stock #000924) females were crossed with C3H/HeSnJ (Jax stock #000661) males to produce B6EiC3SnF1/J litters. Male offspring from these crosses were set up in breeding pairs with B6EiC3Sn *a/A*-Ts(17^16^)65Dn/J (Ts65Dn) females (Jax stock #001924) to give litters containing wildtype (2N) and trisomic pups. Genomic DNA was extracted from ear notch or tail biopsy tissue using the HotSHOT technique [39] for genotyping, and trisomy carriers were detected by chromosome breakpoint polymerase chain reaction [40]. In all cases, animals were culled by UK Animal (Scientific Procedures) Act 1986 Schedule 1 procedures, in accordance with UK Home Office Guidelines. All procedures were approved by the UK Home Office under Project Licence 60/4290.

### Cell culture and transfection

Neuronal cultures were generated in parallel from up to four pups from Ts65Dn litters, and biopsy tissue from each individual animal was retained for genotyping. Cultures of hippocampal neurons were made from pups on the day of birth (P0) as previously described [41], then transfected using Lipofectamine 2000 at 7 days in vitro (DIV), and used in experiments from 13–16 DIV. Cultures of cerebellar granule neurons (CGN) were made from pups of either sex aged between P5 –P7. Pups were culled, and the cerebellum dissected and digested with 0.025% trypsin in Ca^2+^/Mg^2+^ free Hanks Balanced Salt Solution supplemented with 10 mM HEPES pH 7.3 for 20 min at 37°C. Digested tissue was washed in Dulbecco's Modified Eagle Medium supplemented with 10% foetal bovine serum, 60 U/ml DNase and 1% penicillin/streptomycin, and triturated in the same medium to obtain a single cell suspension. Cells were diluted to the required density in Dulbecco's Modified Eagle Medium supplemented with 10% foetal bovine serum and 1% penicillin/streptomycin, and plated in 75 μl spots on 25 mm glass coverslips coated with 50 μg/ml poly-D-lysine. After 1 hour coverslips were flooded with Neurobasal medium supplemented with 2% B27, 0.5 mM L-glutamine, an additional 20 mM KCl (total concentration 25mM) and 1% penicillin/streptomycin. The following day the medium was further supplemented with 2 μM cytosine β-D-arabinofuranoside. Cultures were used for experiments after 8–10 DIV.

### Dynamin I phosphorylation assays

Cultures of CGNs were repolarised for 10 min at room temperature in incubation buffer containing (in mM): 170 NaCl, 3.5 KCl, 0.4 KH_2_PO_4_, 20 TES (N-[tris(hydroxymethyl)methyl]-2-aminoethanesulfonic acid), 5 NaHCO_3_, 5 glucose, 1.2 Na_2_SO_4_, 1.2 MgCl_2_, 1.3 CaCl_2_, pH 7.4, then rapidly lysed by boiling in sample buffer (67 mM SDS, 2 mM EDTA, 9.3% glycerol, 12% β-mercaptoethanol, 0.02% bromophenol blue, 67 mM Tris, pH 6.8) for analysis of basal phosphorylation. In some experiments cells were depolarised in high KCl buffer (incubation buffer with 50 mM KCl exchanged for 50 mM NaCl), or depolarised then left to repolarise for 10 min in incubation buffer, before lysis.

### Preparation of synaptosomes

Synaptosomes were prepared from adult female wildtype and trisomic Ts65Dn mice (11 weeks old). Mice were culled by cervical dislocation and the forebrain was rapidly dissected out and homogenised with a Dounce homogeniser in ice-cold homogenisation buffer containing (in mM): 320 sucrose, 1 EDTA, 5 Tris, pH 7.4. The homogenate was initially centrifuged at 900 × g for 10 min at 4°C, and the supernatant (S1) was further centrifuged at 20200 × g for 10 min at 4°C to give a pellet (P2) enriched in synaptosomes. The synaptosomes were resuspended in ice cold HEPES buffered Krebs containing (in mM): 118.5 NaCl, 4.7 KCl, 1.18 MgSO_4_, 10 glucose, 1 Na_2_HPO_4_, 20 HEPES, pH 7.4, then recentrifuged at 20200 × g for 10 min at 4°C, and resuspended in HEPES buffered Krebs supplemented with 1.3 mM CaCl_2_. The synaptosomes were incubated at 37°C for 10 min and then pelleted at 15900 × g for 2 min and rapidly lysed by boiling in sample buffer. Protein concentration from each synaptosome preparation was estimated by Bradford Assay.

### Western blotting

Samples in lysis buffer were separated by SDS-PAGE and transferred to a nitrocellulose membrane. Blots from CGN lysates were blocked and probed with primary antibodies, washed extensively, probed with HRP conjugated secondary antibodies, then washed again. Antibody binding was detected by enhanced chemiluminescence and visualised with film. Blots from synaptosome samples were prepared in the same way, but probed with IRDye conjugated secondary antibodies and scanned using an LI-COR Odyssey 9120 scanner (LI-COR Biosciences). For all experiments, signals were quantified by densitometry using FIJI and normalised to a β-actin loading control on the same blot prior to further analysis.

### Fluorescence imaging of syp-pHluorin and dextran uptake

All fluorescence imaging was performed on a Zeiss Axio Observer D1 epifluorescence microscope, equipped with a 40× 1.3NA EC Plan Neofluar oil immersion objective and Hamamatsu Orca-ER camera. For pHluorin imaging, coverslips of cultured hippocampal neurons transfected with syp-pHluorin were mounted in an imaging chamber with embedded parallel platinum electrodes (Warner RC-21BRFS), perfused at room temperature with imaging buffer containing (in mM): 119 NaCl, 2.5 KCl, 2 CaCl_2_, 2 MgCl_2_, 25 HEPES, 30 glucose, 10 μM CNQX and 50 μM AP5, adjusted to pH 7.4. In experiments to measure SV pool size, the imaging buffer also contained 1 μM Bafilomycin A1 to inhibit SV acidification. Syp-pHluorin fluorescence was imaged using 480 nm excitation and 510 nm long pass emission filters. Neurons were electrically stimulated at 10 Hz with 1 ms pulses of 100 mA using a Digitimer D330 MultiStim stimulator. At the end of each experiment, cultures were perfused with NH_4_Cl buffer (imaging buffer with 50 mM NH_4_Cl exchanged for 50 mM NaCl) to deacidify all SVs and reveal maximal syp-pHluorin fluorescence. Images were acquired at 4 s intervals and processed using FIJI [42]. Regions of interest were placed over presynaptic terminals that responded to action potential stimulation, and the fluorescence was measured using the Time Series Analyser plugin. Fluorescence data were normalised to the maximal fluorescence obtained in NH_4_Cl buffer to allow comparison between experiments.

Uptake of fluorescent dextran was measured and analysed as described previously [43]. In brief, CGN cultures were repolarised for 10 min in incubation buffer, then electrically stimulated as indicated in the presence of 50 μM tetramethylrhodamine-dextran (TMR-dextran, 40 kDa, Life Technologies). Cells were washed to remove uninternalised TMR-dextran, and 10 fields of view were captured from each coverslip using 550 nm excitation and 630/60 nm band pass emission filters. Images were processed in FIJI using the Max Entropy thresholding algorithm, and the number of fluorescent TMR-dextran puncta between 0.25–0.88 μm^2^ counted using the Analyse Particles tool. Counts were corrected for non-specific background binding by subtracting the number of puncta measured in unstimulated control coverslips from each genotype.

### Transmission electron microscopy of HRP uptake and endosome budding

Cultures of CGNs were repolarised for 10 min in incubation buffer, then stimulated with 400 action potentials at 40 Hz in the presence of 10 mg/ml horse-radish peroxidase (HRP, Sigma-Aldrich). Immediately following the end of stimulation, cultures were washed to remove uninternalised HRP and then fixed with 2% glutaraldehyde in phosphate buffered salt solution. After washing in 100 mM Tris (pH 7.4), HRP was developed with 0.1% w/v 3,3'-diaminobenzidine and 0.2% v/v hydrogen peroxide. After further washes in 100 mM Tris, cultures were stained with 1% v/v osmium tetroxide for 30 min and processed for transmission electron microscopy (TEM) as described previously [44].

SV budding from bulk endosomes labelled with HRP was measured and analysed as described previously [45]. Briefly, cultures of CGNs were repolarised for 10 min in incubation buffer, then depolarised in high KCl buffer containing 10 mg/ml HRP for 2 min. Cultures were then washed to remove uninternalised HRP, allowed to recover in incubation buffer for 2 min, and then immediately depolarised again twice for 30 sec in high KCl buffer with a 30 sec interval to unload HRP labelled SVs. Finally, cultures were left to rest for 30 min in incubation buffer to allow endosome budding to proceed. Samples were fixed at each of the load, unload and rest steps, then developed and processed for transmission electron microscopy as above. Images were acquired on an FEI Tecnai 12 transmission electron microscope, and analysed in FIJI. HRP labelled structures with a diameter ≥100 nm were arbitrarily classified as endosomes. Results are presented as HRP-labelled structures per nerve terminal cross section for both SVs and bulk endosomes.

### Statistical analysis

All data analysis was performed in Microsoft Excel and GraphPad Prism 6. Two-tailed unpaired t-tests were used to compare two groups, one-way ANOVA with Holm-Šídák post-hoc tests were used to compare more than two groups, and two-way ANOVA with Holm-Šídák post-hoc tests were used to compare binned frequency distributions between groups. In all analyses, the sample size (n) was taken to be the number of independent experiments. All data are presented as mean values ± standard error of the mean (SEM).

## Results

**Fig 1:**
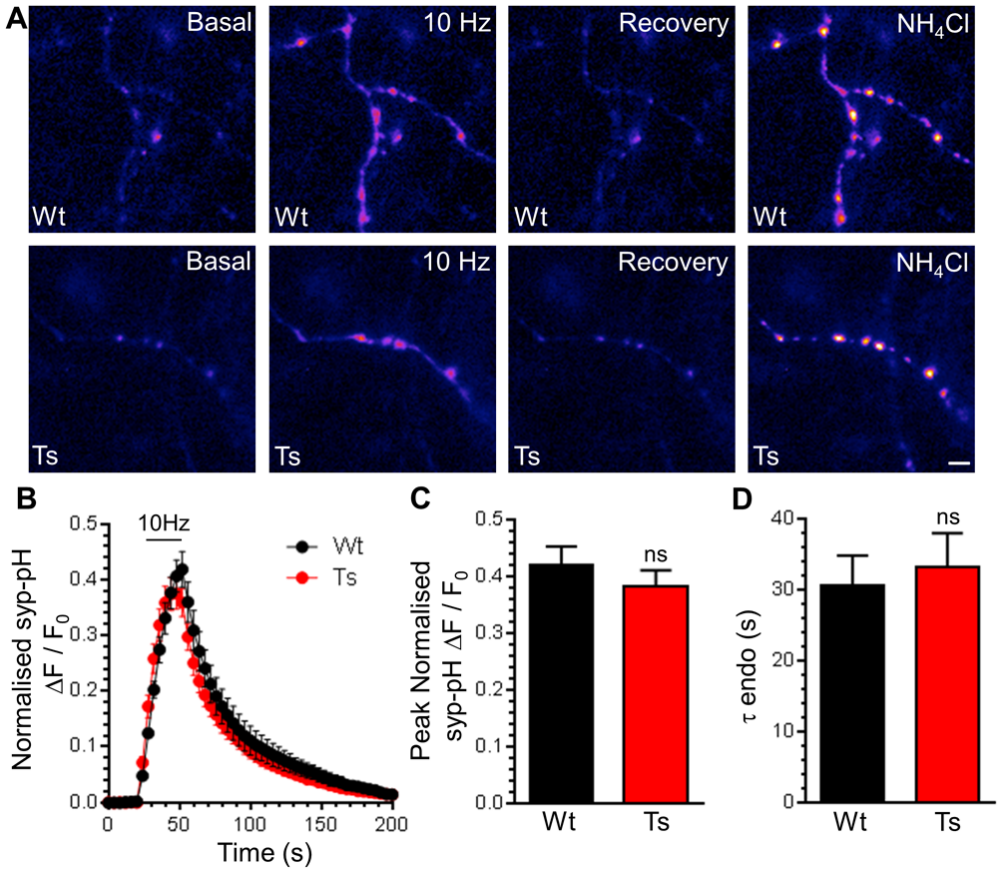
Ts65Dn trisomy does not affect SV exocytosis or CME. A) Primary cultures of hippocampal neurons expressing syp-pHluorin (syp-pH) from either trisomic (Ts) or wild-type (Wt) littermates were stimulated with a train of 300 action potentials (10 Hz). Cultures were when left to recover before a pulse of NH_4_Cl buffer was applied. Representative false colour images display a transfected neuron (Wt or Ts) either at rest (Basal), during stimulation (10 Hz), 2 min after stimulation (Recovery) and during challenge with NH_4_Cl buffer. Scale bar represents 3 μm. B) Mean fluorescence traces ± SEM (ΔF/F_0_) normalised to peak fluorescence obtained in NH_4_Cl buffer (black circles, Wt; red circles Ts). Stimulation is indicated by bar. C) Mean peak normalised syp-pH fluorescence during stimulation ± SEM. D) Mean time constant (τ) for post-stimulation fluorescence recovery ± SEM (n = 5 Wt, n = 9 Ts, two-tailed unpaired t-test, ns = p > 0.05).

Enlarged endosomes are present in the neurons of DS patients and Ts65Dn mice [7,32] and a number of endocytosis genes are part of the DS trisomy. We therefore investigated whether altered SV recycling may underlie the synaptic defects observed in the Ts65Dn mouse. We first determined whether activity-dependent SV exocytosis or endocytosis was perturbed in primary hippocampal cultures derived from either trisomic mice or wild-type littermate controls. SV turnover was monitored using the genetically-encoded fluorescent reporter synaptophysin-pHluorin (syp-pHluorin) [20]. The fluorescent pHluorin moiety is exquisitely pH-sensitive, and when fused within an intraluminal loop of the SV protein synaptophysin is quenched by the acidic SV environment. During SV exocytosis syp-pHluorin is exposed to the neutral extracellular environment and its fluorescence is unquenched. It is then retrieved via SV endocytosis and the reformed SVs are rapidly acidified, quenching the reporter. SV acidification occurs with faster kinetics than endocytosis [46], and therefore the recovery kinetics of the syp-pHluorin response after stimulation provides a readout of SV endocytosis rate. Cultures were stimulated with a train of 300 action potentials (10 Hz), which evoked a characteristic increase in syp-pHluorin fluorescence, followed by a recovery to baseline on termination of stimulation (Fig 1A and 1B). The peak syp-pHluorin response in trisomic neurons was not significantly different to wild-type (Fig 1C), suggesting that there was no obvious difference in the extent of SV exocytosis. Furthermore the time constant for syp-pHluorin fluorescence recovery after stimulation was also not significantly different between genotypes (Fig 1D). This suggests CME is unaffected in trisomic neurons, since it is the dominant SV endocytosis mode under these stimulation conditions [47].

**Fig 2:**
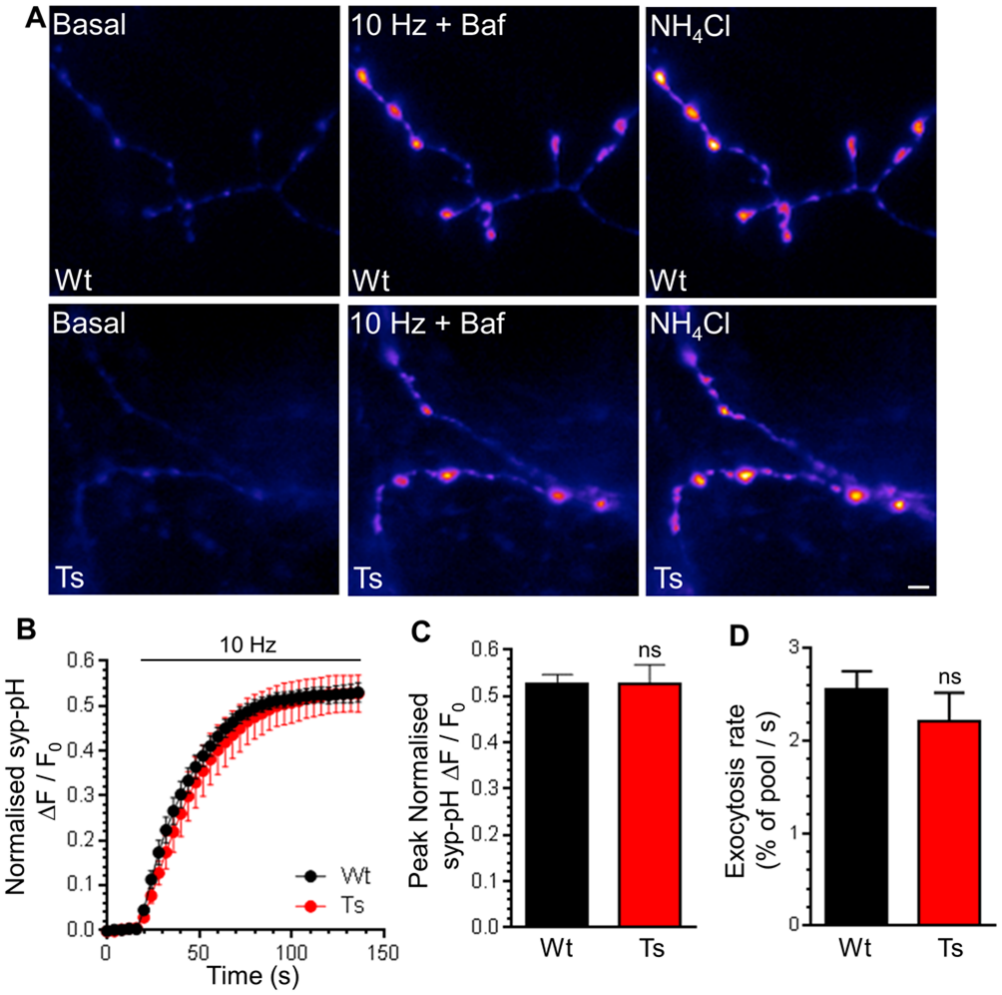
Ts65Dn trisomy does not affect the SV recycling pool. A) Primary cultures of hippocampal neurons expressing syp-pHluorin (syp-pH) from either trisomic (Ts) or wild-type (Wt) littermates were stimulated with a train of 1200 action potentials (10 Hz) in the presence of Bafilomycin A1 (Baf) before a pulse of NH_4_Cl buffer was applied. Representative false colour images display a transfected neuron (Wt or Ts) either at rest (Basal), during stimulation (10 Hz + Baf) or during challenge with NH_4_Cl buffer. Scale bar represents 2 μm. B) Mean fluorescence traces ± SEM (ΔF/F_0_) were normalised to peak fluorescence obtained in NH_4_Cl buffer (black circles, Wt; red circles Ts). C) Mean peak normalised syp-pH fluorescence during stimulation ± SEM. D) Mean initial rate of syp-pHluorin increase on stimulation ± SEM (n = 5 for both genotypes, two-tailed unpaired t-test, ns = p > 0.05).

CaN activity positively regulates the size of the SV recycling pool, defined as SVs that are mobilised during action potential stimulation [22]. Since increased expression of RCAN1 in Ts65Dn neurons [48] may alter CaN activity, we determined the size of the recycling pool. To determine the recycling pool size, SV acidification in syp-pHluorin expressing neurons was acutely blocked with Bafilomycin A1. This reveals the cumulative number of SVs that visit the plasma membrane during a prolonged train of 1200 action potentials (10 Hz). In wild-type neurons, action potential stimulation evoked a fluorescence increase that saturated over time, indicating that the full SV recycling pool was mobilised (Fig 2A). Trisomic neurons displayed an almost identical response (Fig 2A and 2B), indicating that either the size of the total SV recycling pool (Fig 2C) or the rate at which it is mobilised (Fig 2D) is unaffected by the presence of the DS trisomy.

**Fig 3:**
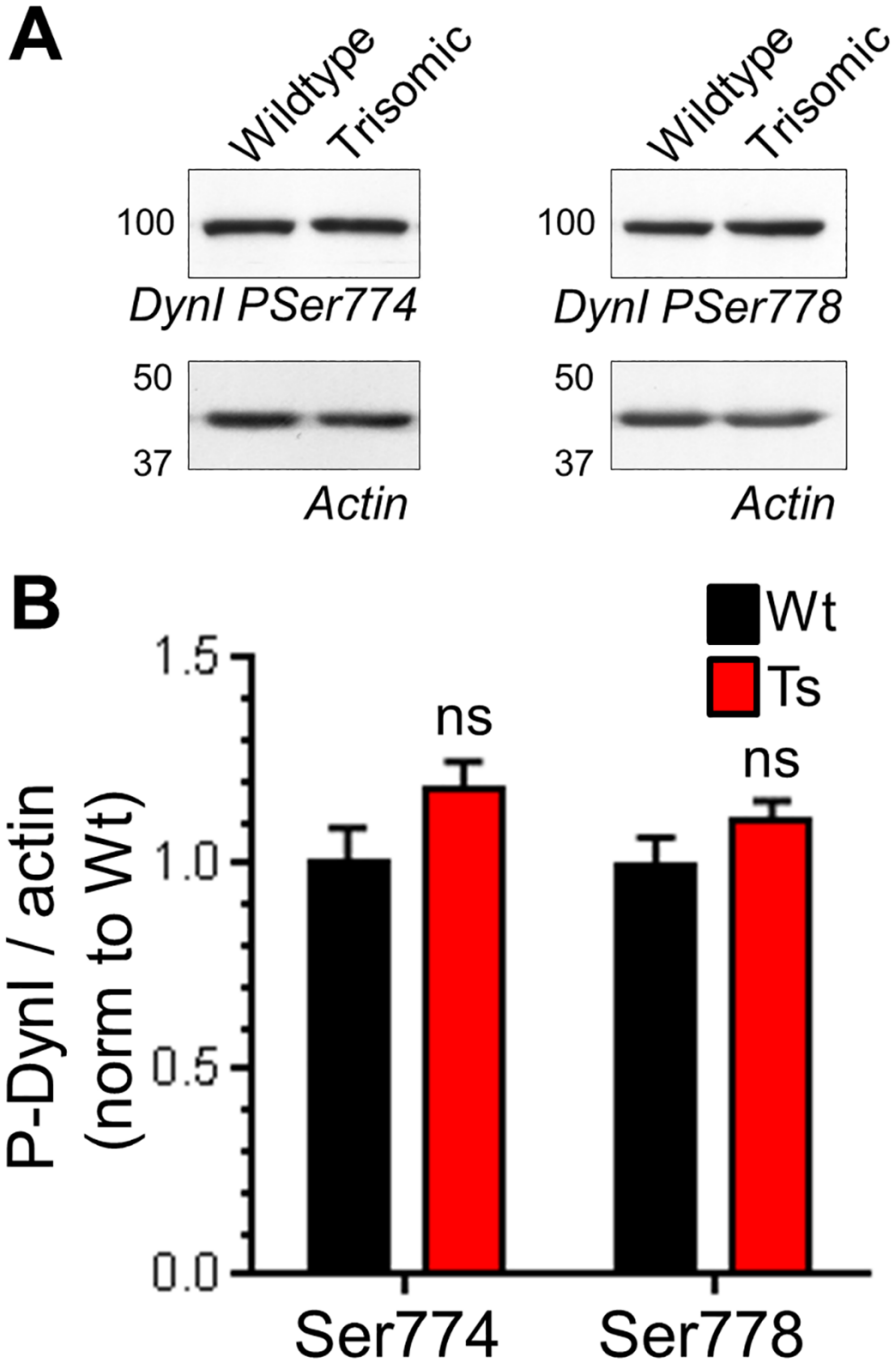
Ts65Dn trisomy has no effect on basal phosphorylation of dynamin I. Wild-type (Wt) and trisomic (Ts) CGNs were lysed and the phosphorylation status of either Ser774 (PSer774) or Ser778 (PSer778) on dynamin I (DynI) was monitored by western blotting with phospho-specific antibodies. A) Representative blots are displayed from Wt and Ts CGN lysates probed with either PSer774 or PSer778 antibodies and actin loading controls. B) Mean phosphorylation levels of either PSer774 or PSer778 normalised to actin are displayed in either Wt (black bars) or Ts (red bars) CGNs ± SEM. Data are presented normalised to Wt (n = 10 PSer774; n = 11 PSer778, two-tailed unpaired t-test, ns = p > 0.05.

**Fig 4:**
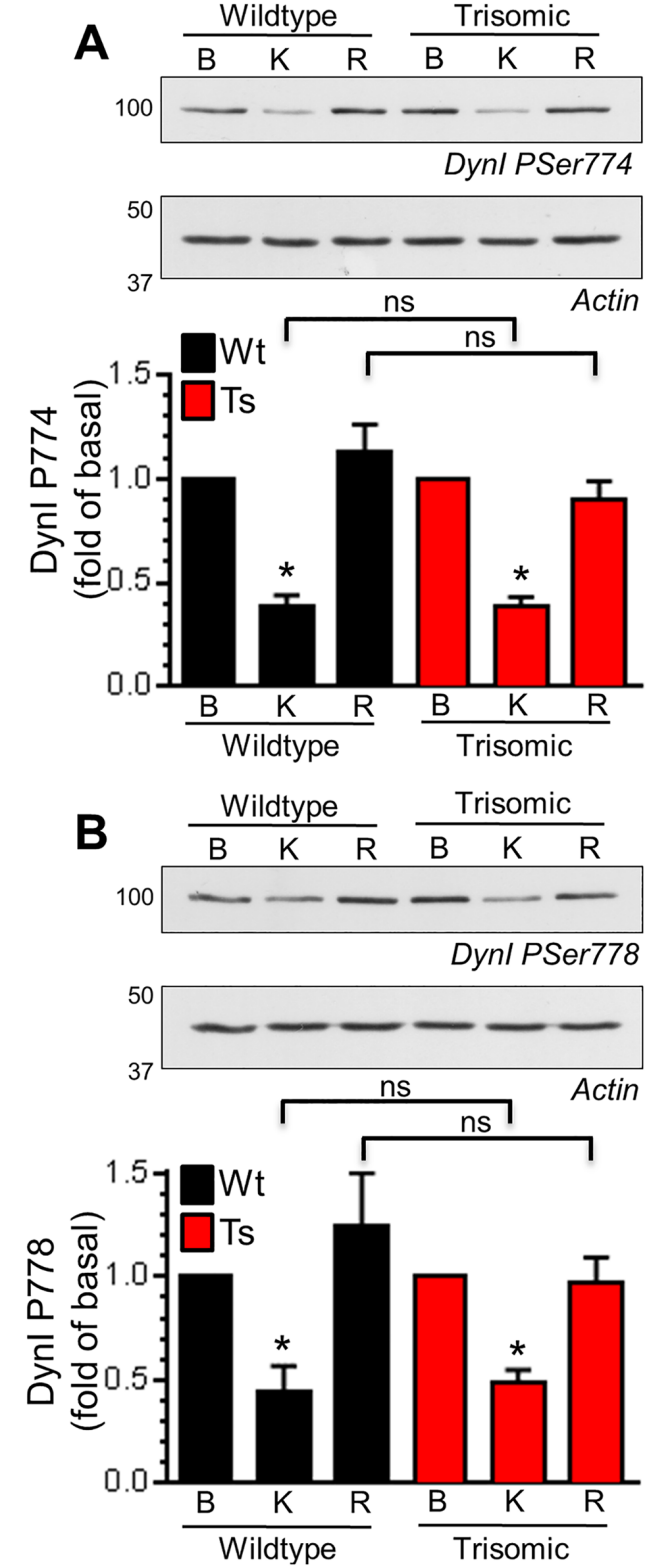
Ts65Dn trisomy has no effect on activity-dependent phosphorylation of dynamin I. Wild-type (Wt) and trisomic (Ts) CGNs were lysed either in basal conditions (B), after 30 s depolarisation with 50 mM KCl (K) or after 10 min of repolarisation (R). The phosphorylation status of either Ser774 (PSer774, A) or Ser778 (PSer778, B) on dynamin I (DynI) was monitored by western blotting with phospho-specific antibodies. Representative blots are displayed from Wt and Ts CGN lysates probed with either PSer774 or PSer778 antibodies and actin loading controls. Mean phosphorylation levels of either PSer774 (A) or PSer778 (B) normalised to actin and basal phosphorylation are displayed in either Wt (black bars) or Ts (red bars) CGNs ± SEM (n = 4 PSer774; n = 3 PSer778, one-way ANOVA with Holm-Šídák, ns = p > 0.05.

The presence of enlarged endosomes in both DS patients and Ts65Dn mice suggested a potential defect in ADBE, since this endocytosis mode traffics SV cargo and membrane via endosomal intermediates [21]. In support, CaN activity is required for triggering of ADBE [24,25]. We first determined the effect of DS trisomy on the phosphorylation status of Ser774 and Ser778 on dynamin I, since CaN-dependent dephosphorylation of these residues is essential for ADBE triggering [24]. We performed these experiments in primary cultures of CGNs, since ADBE is best characterised in this system. The phosphorylation levels of either Ser774 or Ser778 were not significantly different between resting wild-type and trisomic neurons when assessed by western blotting with phospho-specific antibodies (Fig 3). Similar results were also obtained from isolated nerve terminals (synaptosomes) prepared from adult mice (Fig A in S1 File). We next tested whether activity-dependent changes in dynamin I phosphorylation status were affected by the presence of the Ts65Dn trisomy. Dynamin I was significantly dephosphorylated on both Ser774 and Ser778 during a hyperkalaemic depolarising stimulus in wild-type CGNs, and was fully rephosphorylated during a 10 minute rest period (Fig 4). Dynamin I displayed a very similar phosphorylation profile in trisomic CGNs (Fig 4). This absence of effect on dynamin I phosphorylation was not due to alterations in total dynamin I, since this was unaffected by both stimulation and genotype (Fig B in S1 File). Therefore activity-dependent dephosphorylation of dynamin I by CaN and its rephosphorylation after stimulation was unaffected by the Ts65Dn trisomy.

**Fig 5:**
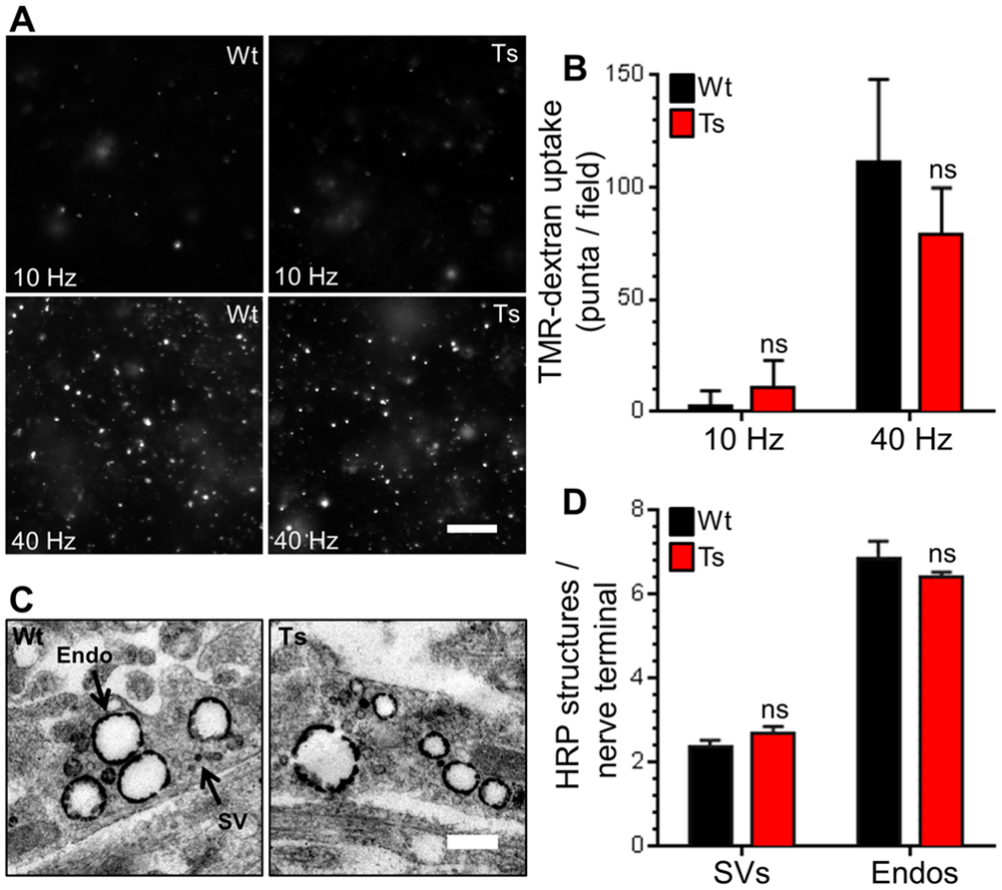
Ts65Dn trisomy has no effect on ADBE. *A*,B) Wild-type (Wt) and trisomic (Ts) CGNs were challenged with trains of action potentials (either 10 Hz 20 s or 40 Hz 10 s) in the presence of 50 μM TMR-dextran. TMR-dextran was immediately washed away on termination of stimulation. A) Representative images of TMR-dextran uptake are displayed, scale bar indicates 20 μm. B) Mean number of TMR-dextran puncta detected per field of view in each condition for either Wt (black bars) or Ts (red bars) neurons ± SEM (n = 6, Wt 10 Hz; n = 10, Wt 40 Hz; n = 5, Ts 10 Hz; and n = 10, Ts 40 Hz) independent experiments for each condition, one-way ANOVA with Holm-Šídák, ns = p > 0.05. C, D) CGNs were challenged with a train of 400 action potentials (40 Hz) in the presence of 10 mg/ml HRP. C) Representative images show a typical HRP-labelled endosome (Endo) and synaptic vesicle (SV) in either Wt or Ts nerve terminals. Scale bar represents 500 nm. D) Mean number of HRP-labelled endosomes and SVs per nerve terminal for either Wt (black bars) or Ts (red bars) neurons is displayed ± SEM (n = 3 independent experiments for each genotype, two-tailed unpaired t-tests, ns = p > 0.05).

The lack of effect on CaN-mediated dynamin I dephosphorylation suggested that ADBE may be unaltered in trisomic neurons. We determined this directly by monitoring uptake of the large (40 kDa) fluid phase marker TMR-dextran. TMR-dextran selectively reports ADBE since its size prevents its accumulation into SVs retrieved via CME [47]. Delivery of a high frequency train of 400 action potentials (40 Hz) evoked a robust increase in TMR-dextran uptake in wild-type CGNs (Fig 5A and 5B). A train of lower frequency action potentials (10 Hz for 20 sec) failed to evoke any TMR-dextran uptake, confirming activity-dependent triggering of ADBE [47]. When these experiments were repeated in trisomic CGNs, no differences in TMR-dextran uptake were observed when compared to wild-type (Fig 5A and 5B), suggesting that the Ts65Dn trisomy has no effect on either the triggering threshold or overall extent of ADBE.

To confirm that both CME and ADBE were unaffected in trisomic neurons, we also examined uptake of the fluid phase marker HRP at the ultrastructural level. This assay provides straightforward discrimination of CME and ADBE via the generation of HRP-labelled SVs and endosomes respectively [47]. CGNs were challenged with a train of intense neuronal activity (40 Hz 10 s) in the presence of HRP and then immediately fixed. The number of HRP-labelled SVs or endosomes per nerve terminal were not significantly different between genotypes (Fig 5C and 5D). The lack of phenotype was not due to changes in nerve terminal area, since this parameter was also unchanged (Fig C in S1 File). This confirms that both CME and ADBE were unaffected by the Ts65Dn trisomy.

**Fig 6:**
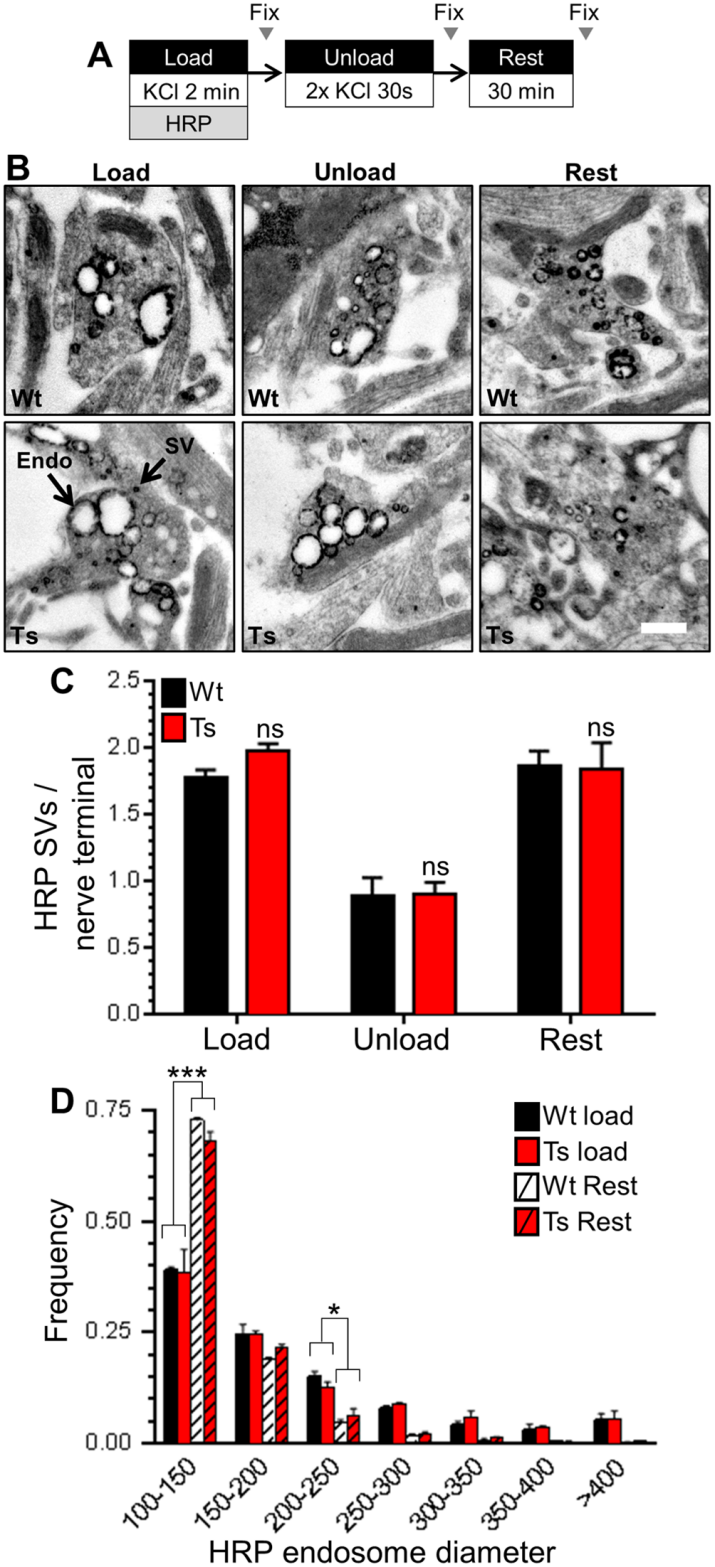
Ts65Dn trisomy has no effect on SV generation from bulk endosomes. *A)* Wild-type (Wt) and trisomic (Ts) CGNs were stimulation with 50 mM KCl for 2 min in the presence of 10 mg/ml HRP. After 2 min CGNs were challenged with 2 consecutive 30 s KCl stimuli (50 mM) and then left to rest for 30 min. CGNs were fixed either immediately after loading (Load), after the two consecutive KCl stimuli (Unload) or after the 30 min rest period (Rest). B) Representative images show a typical HRP-labelled endosome (Endo) and synaptic vesicle (SV) in either Wt or Ts nerve terminals in either Load, Unload or Rest conditions. Scale bar represents 500 nm. C) Mean number of HRP-labelled SVs per nerve terminal for either Wt (black bars) or Ts (red bars) neurons is displayed for either the Load, Unload or Rest conditions ± SEM (n = 3 coverslips for each genotype, one-way ANOVA with Holm-Šídák, ns = p > 0.05). D) Frequency histogram displaying the diameter of HRP-labelled endosomes in either Wt or Ts nerve terminals at either the Load or Rest conditions (n = 3 independent experiments for each genotype, two-way ANOVA with Holm-Šídák, *** = p < 0.001, * = p < 0.05, all other conditions ns = p > 0.05).

Finally, we tested whether SV generation from bulk endosomes was affected by the DS trisomy, since this process also requires CaN activity [26]. We used a characterised morphological assay [49] that tracks the fate of SVs generated from bulk endosomes. In this assay CGNs are challenged with intense stimulation in the presence of HRP, generating HRP-labelled SVs and endosomes. CGNs are then immediately challenged with an identical stimulus to selectively deplete HRP-labelled SVs formed by CME. CGNs are left to recover, following which the number of new HRP-labelled SVs that are generated from bulk endosomes can be quantified (Fig 6A and 6B). We observed no difference in either the generation, depletion or reformation of HRP-labelled SVs when wild-type and trisomic CGNs were compared (Fig 6C), suggesting that the Ts65Dn trisomy does not affect SV generation from bulk endosomes. This was confirmed by monitoring the size of HRP-labelled bulk endosomes, since their diameter decreases as they donate membrane to generate new SVs [49]. No genotype-dependent difference in the diameter of HRP-labelled endosomes was observed immediately after HRP loading (Fig 6D). The diameter of HRP-labelled endosomes then decreased to the same extent after the 30 minute rest period in both wild-type and trisomic CGNs, indicating that they had generated a similar number of SVs (Fig 6D). Together these results indicate that SV budding from bulk endosomes formed by ADBE is unaffected by the Ts65Dn trisomy.

## Discussion

A number of genes within the DS trisomy encode proteins that are either implicated or directly involved in the control of SV recycling, suggesting SV recycling could be altered in this condition. In support, neurons derived from mouse models of DS display altered plasticity and ultrastructural changes consistent with altered endocytic or trafficking processes. However, we found that in Ts65Dn mice, key parameters of neuronal SV recycling were indistinguishable from wild-type controls. This suggests that SV recycling may be unaffected in DS and does not contribute to the neurological problems displayed in the condition.

The Ts65Dn mouse is currently the best characterised model of DS, and has been widely used for preclinical testing of drugs to treat learning and memory impairments in DS [50]. It is an imperfect model however, carrying a segmental trisomy of Mmu16 containing orthologues of only 88 of the 157 conserved protein encoding genes found on Hsa21 [30]. Furthermore it carries an additional 60 annotated genes from Mmu17 within its trisomy that do not have orthologues on Hsa21 [51]. Nonetheless, the Ts65Dn mouse displays several important hallmarks of DS, including deficits in learning and memory [38], selective neurodegeneration of cholinergic neurons [52] and the presence enlarged Rab5-positive endosomes within the brain [32,33]. Thus while the Ts65Dn mouse is a reasonable model of DS, we cannot rule out that differences in SV recycling may be present in other mouse models containing trisomies more closely resembling Hsa21 [38].

As stated above the Ts65Dn mouse displays neurodegeneration, in agreement with the increased incidence of Alzheimer’s Disease in DS patients [53]. Presynaptic dysfunction precedes neurodegeneration in a number of preclinical models [54,55] suggesting this may in part precipitate this event. We were unable to determine whether presynaptic dysfunction occurs in aged Ts65Dn mice, since primary neuronal cultures can only be currently prepared from embryonic or neonatal tissue. It is therefore possible that the enlarged Rab5-positive endosomes observed in Ts65Dn neurons may cause presynaptic dysfunction later in life. However it should be considered that DS is primarily a neurodevelopmental disorder, with neurological deficits present from birth.

Among the trisomic Hsa21 gene orthologues the Ts65Dn mouse carries, there are several with potential relevance to SV recycling including *Synj1*, *Itsn1*, *Rcan1*, *App* and *Dyrk1a*. The CaN inhibitor RCAN1 was of particular interest, since RCAN1 is thought to inhibit CaN activity [28,56,57] and CaN activity is required for multiple aspects of the SV life cycle [22–26]. Indeed CaN knockout mice display impaired neurotransmission during intense stimulation and prominent hyperphosphorylation of proteins involved in the SV cycle including dynamin I [58]. CaN knockout mice also show deficits in working memory [59,60], indicating that the differences in neurotransmission in the absence of CaN activity have profound effects on normal cognition. In tissue from DS brains, RCAN1 protein expression is increased [27,28], and over-expression of RCAN1 in secretory cells negatively regulates granule fusion [61] and endocytosis [16]. Furthermore mice over-expressing RCAN1 also show reduced LTP maintenance, accompanied by behavioural deficits in learning and memory [62]. A direct link between RCAN1 expression and CaN activity has been made, with RCAN1 knockout mice displaying increased CaN activity in hippocampal lysates [63]. However, transgenic mice overexpressing RCAN1 display no alterations in hippocampal CaN activity [62]. This suggests that increased *Rcan1* gene dosage such as observed in DS trisomy may be insufficient to inhibit CaN activity, in agreement with our results.

Both intersectin-1 and synaptojanin have established roles in CME at synapses [10,11,64]. Furthermore the protein kinase Dyrk1a phosphorylates both synaptojanin-1 [14,65] and RCAN1 [66], suggesting possible synergistic effects of these trisomic genes [67]. Dyrk1a phosphorylates several other CME proteins including amphiphysin, dynamin, AP180 and α- and β-adaptins [68–70], suggesting it plays a potential regulatory role during CME. Our studies using both the genetically-encoded SV cargo syp-pHluorin, and uptake of fluid phase HRP, have shown that both the kinetics and extent of CME are unaffected in Ts65Dn neurons.

Enlarged Rab5-positive early endosomes are present in neurons from both pre-birth DS patients [7], and Ts65Dn mice [32]. This defect is linked to cholinergic neuron degeneration due to NGF transport failure [33] and altered synaptic neurotrophin signalling [71]. APP plays a key role in the formation of these enlarged endosomes, since normalisation to two copies of APP in trisomic mice stops their formation. However, APP over-expression alone is not sufficient to induce endosomal pathology, showing that other trisomic genes must contribute to the effect [32]. One candidate is synaptojanin-1, since it is essential for the formation of enlarged early endosomes in lymphoblastoid cell lines derived from DS patients [72]. Since synaptojanin also plays a key role in SV endocytosis, this presents a potential cross-over between DS endosomal pathology and SV recycling pathways. In addition, accumulating evidence suggest that classical endosomal trafficking pathways may intersect with multiple SV endocytosis modes [8] suggesting the pathogenic processes that lead to enlarged early endosomes could either feed through to, or originate at, the presynapse. However the fact that that CME, ADBE and SV generation from bulk endosomes are unaffected by trisomy in Ts65Dn neurons, indicates that the acquisition of endosomal pathology and SV recycling pathways are most likely unconnected in DS.

In acute hippocampal slices from Ts65Dn mice, basal transmission appears normal [36], in agreement with our finding that SV exocytosis and recycling pool size is unaffected by the trisomy. In contrast, hippocampal LTP induction is strongly reduced in Ts65Dn mice, which is most likely caused by excessive GABAergic inhibition [35–37,73]. Encouragingly, recent studies have shown that pharmacological antagonism of post-synaptic GABA_A_ receptors can rescue behavioural deficits in learning and memory in Ts65Dn mice [74,75]. Our data reveals an absence of presynaptic dysfunction in the central nerve terminals of Ts65Dn mice, suggesting that future pharmacotherapy should continue to focus on the established alterations in excitatory / inhibitory balance at the circuit level.

## Supporting Information

S1 File **Fig A)** Forebrain synaptosomes from either wild-type (Wt) and trisomic (Ts) mice were lysed and the phosphorylation status of either Ser774 (PSer774) or Ser778 (PSer778) on dynamin I (DynI) was monitored by western blotting with phospho-specific antibodies. Representative blots are displayed from Wt and Ts synaptosome lysates probed with either PSer774 or PSer778 antibodies and actin loading controls. Mean phosphorylation levels of either Ser774 or Ser778 normalised to actin are displayed in either Wt (black bars) or Ts (red bars) synaptosome lysates ± SEM. Data are presented normalised to Wt (n = 3 animals for each genotype, two-tailed unpaired t-test, ns = p > 0.05). **Fig B)** Wild-type (Wt) and trisomic (Ts) CGNs were lysed either in basal conditions, after 30 s depolarisation with 50 mM KCl. The effect of genotype or stimulation on total dynamin I (DynI) was monitored by western blotting with dynamin antibodies. Representative blots are displayed from Wt and Ts CGN lysates probed with DynI antibodies and actin loading controls. Mean DynI levels normalised to actin are displayed in either Wt (black bars) or Ts (red bars) CGNs ± SEM (n = 3), one-way ANOVA with Holm-Šídák, ns = p > 0.05. **Fig C)** CGNs were challenged with a train of 400 action potentials (40 Hz) in the presence of 10 mg/ml HRP. The mean nerve terminal area (μm^2^) is displayed in either wild-type (Wt, black bars) or trisomic (Ts, red bars) neurons ± SEM (n = 3 coverslips for each genotype with 50 nerve terminals examined per coverslip). **Fig D)** Uncropped original Western blots from Fig 3. **Fig E)** Uncropped original Western blots from Fig 4. **Fig F)** Uncropped original Western blots from Fig A. **Fig G)** Uncropped original Western blots from Fig B.
(PDF)

## Abbreviations

ADBE: activity dependent bulk endocytosis
CGN: cerebellar granule neurons
CME: clathrin-mediated endocytosis
DIV: days in vitro
DS: Down syndrome
HRP: horseradish peroxidase
LTP: long-term potentiation
SV: synaptic vesicle
TMR: tetramethylrhodamine
Hsa21: human chromosome 21
Mmu16: mouse chromosome 16
SEM: standard error of the mean
