# Supplementary material for: Synaptic Vesicle Recycling Is Unaffected in the Ts65Dn Mouse Model of Down Syndrome
Source: PLoS One. 2016 Jan 25;11(1):e0147974. doi: 10.1371/journal.pone.0147974 (PMC4726538; doi:10.1371/journal.pone.0147974)
Supplement: S1 File — Fig A) Forebrain synaptosomes from either wild-type (Wt) and trisomic (Ts) mice were lysed and the phosphorylation status of either Ser774 (PSer774) or Ser778 (PSer778) on dynamin I (DynI) was monitored by western blotting with phospho-specific antibodies. Representative blots are displayed from Wt and Ts synaptosome lysates probed with either PSer774 or PSer778 antibodies and actin loading controls. Mean phosphorylation levels of either Ser774 or Ser778 normalised to actin are displayed in either Wt (black bars) or Ts (red bars) synaptosome lysates ± SEM. Data are presented normalised to Wt (n = 3 animals for each genotype, two-tailed unpaired t-test, ns = p > 0.05). Fig B) Wild-type (Wt) and trisomic (Ts) CGNs were lysed either in basal conditions, after 30 s depolarisation with 50 mM KCl. The effect of genotype or stimulation on total dynamin I (DynI) was monitored by western blotting with dynamin antibodies. Representative blots are displayed from Wt and Ts CGN lysates probed with DynI antibodies and actin loading controls. Mean DynI levels normalised to actin are displayed in either Wt (black bars) or Ts (red bars) CGNs ± SEM (n = 3), one-way ANOVA with Holm-Šídák, ns = p > 0.05. Fig C) CGNs were challenged with a train of 400 action potentials (40 Hz) in the presence of 10 mg/ml HRP. The mean nerve terminal area (μm2) is displayed in either wild-type (Wt, black bars) or trisomic (Ts, red bars) neurons ± SEM (n = 3 coverslips for each genotype with 50 nerve terminals examined per coverslip). Fig D) Uncropped original Western blots from Fig 3. Fig E) Uncropped original Western blots from Fig 4. Fig F) Uncropped original Western blots from Fig A. Fig G) Uncropped original Western blots from Fig B. (PDF) [file pone.0147974.s001.pdf]

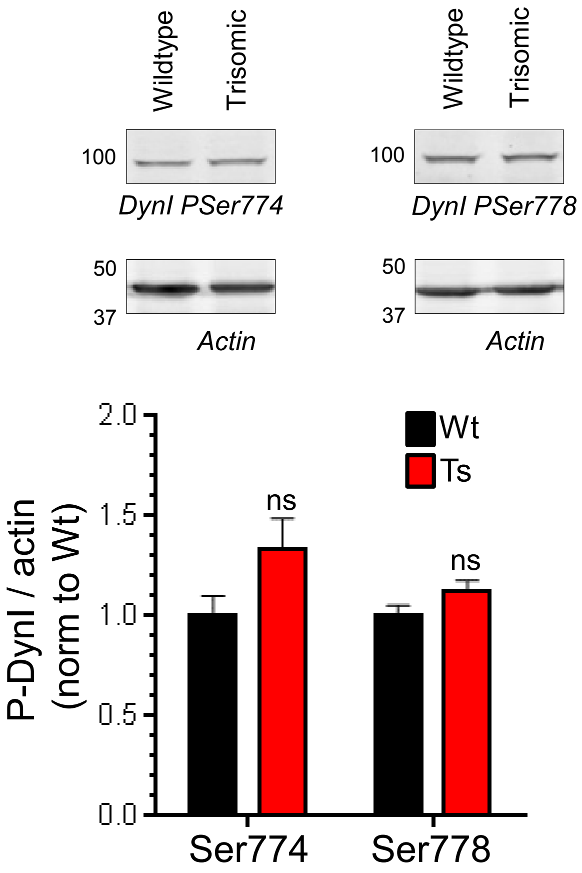

Supplementary Figure A

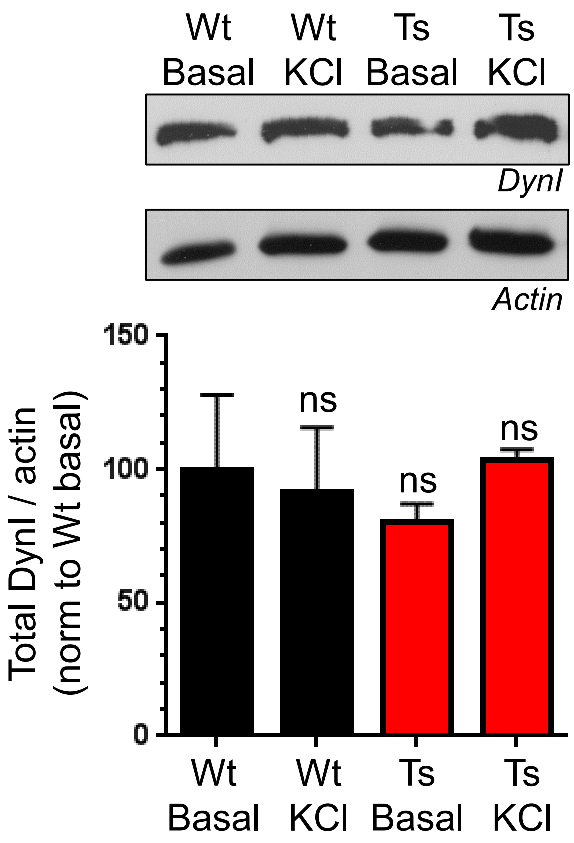

Supplementary Figure B

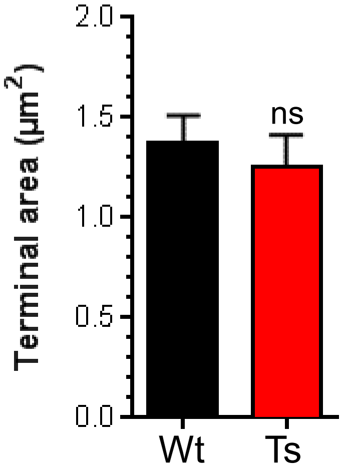

Supplementary Figure C

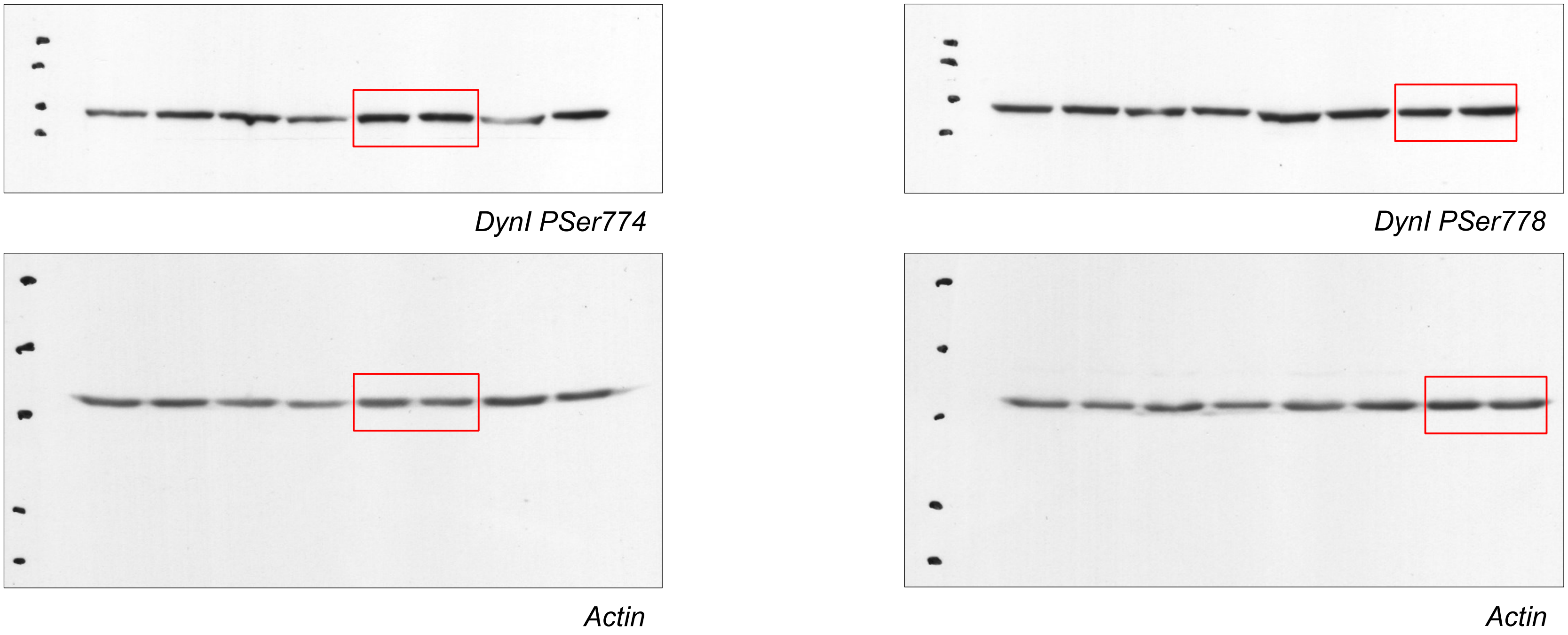

Supplementary Figure D

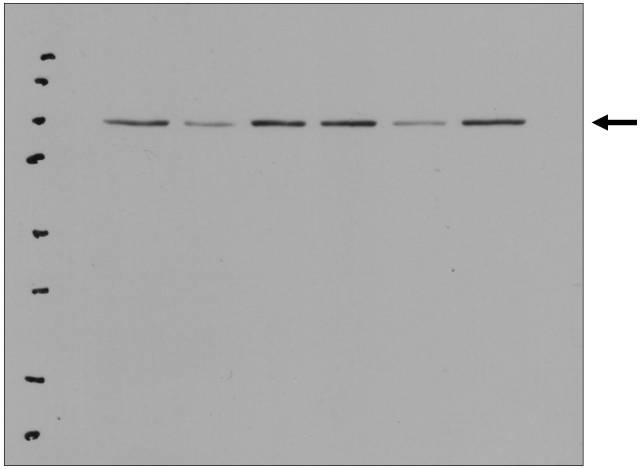

DynI PSer774

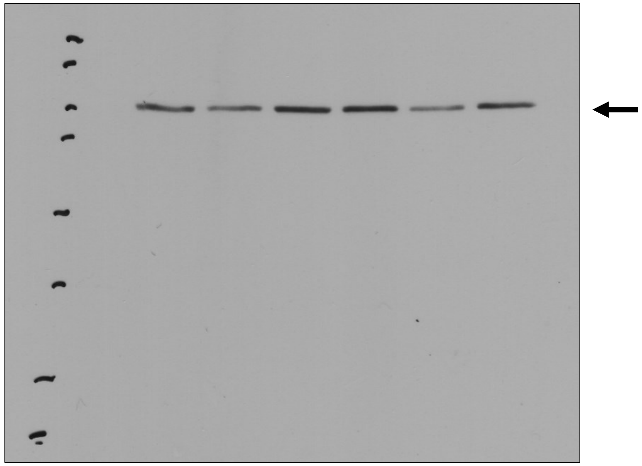

DynI PSer778

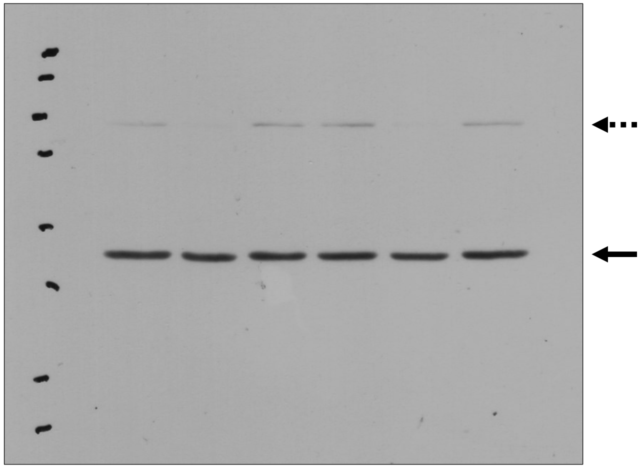

Actin

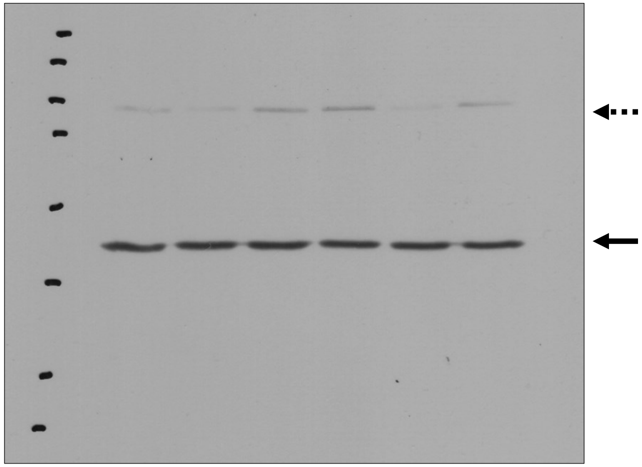

Actin

Supplementary Figure E

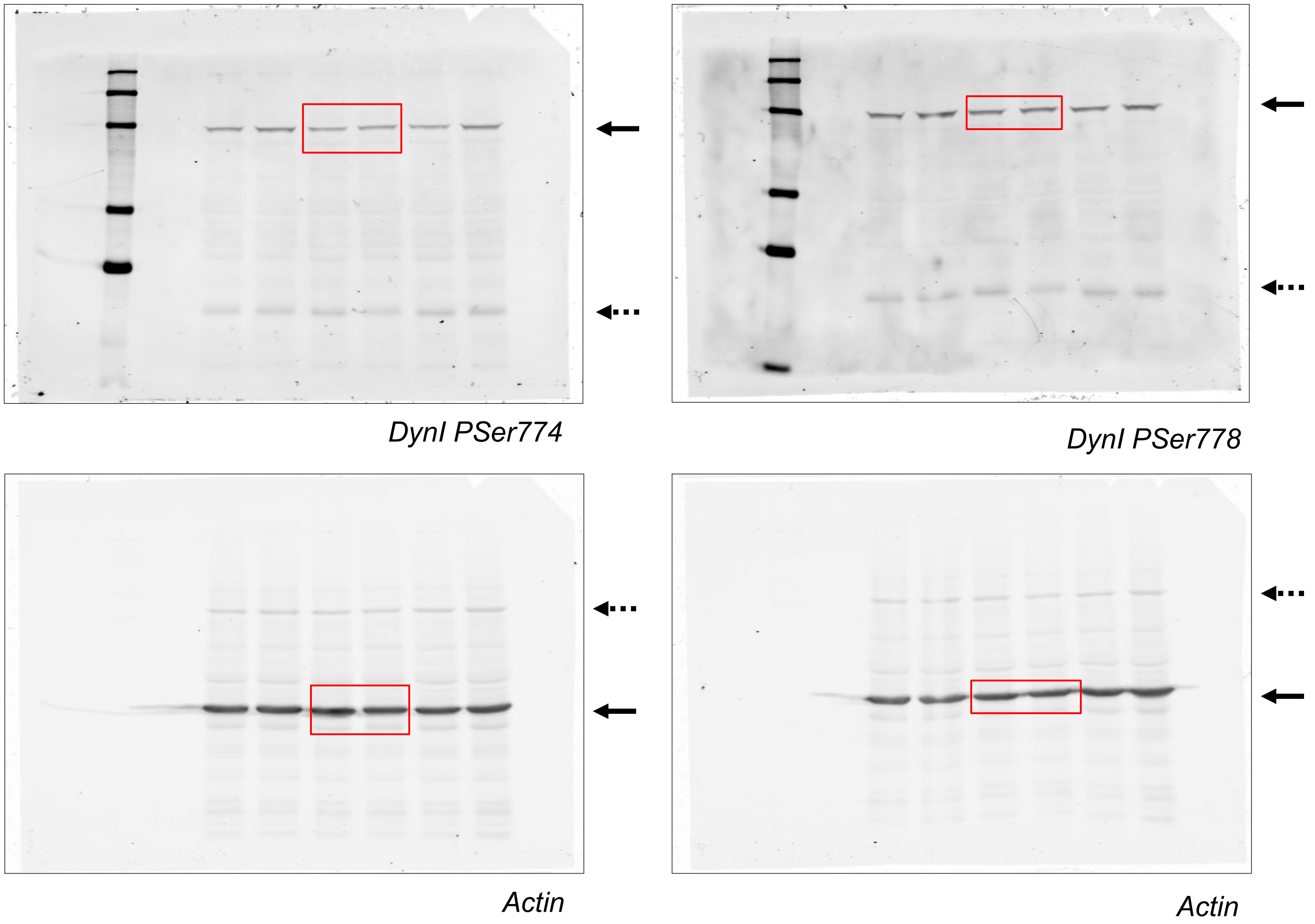

Supplementary Figure F

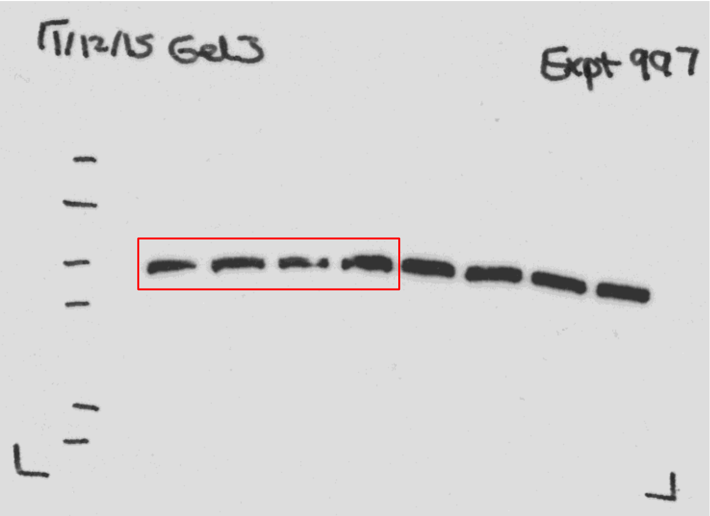

Dynl

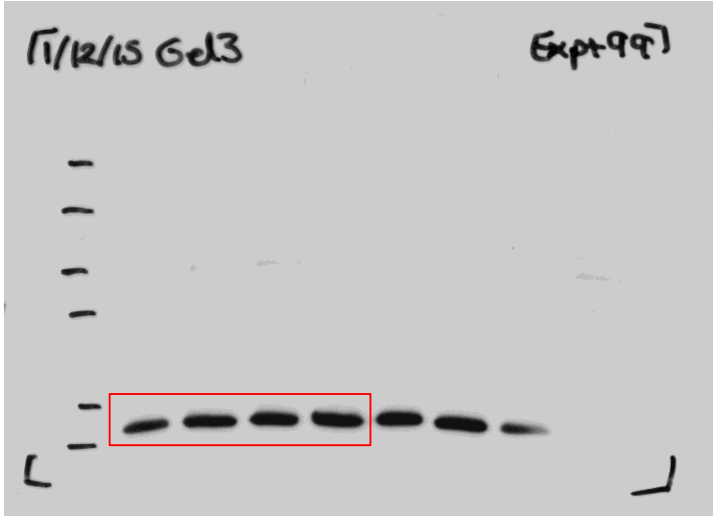

Actin

Supplementary Figure G
